# 5-Fluorotryptamine is a partial agonist at 5-HT_3_ receptors, and reveals that size and electronegativity at the 5 position of tryptamine are critical for efficient receptor function

**DOI:** 10.1016/j.ejphar.2007.11.014

**Published:** 2008-02-12

**Authors:** Kiowa S. Bower, Kerry L. Price, Laura E.C. Sturdee, Mariza Dayrell, Dennis A. Dougherty, Sarah C.R. Lummis

**Affiliations:** a California Institute of Technology, Pasadena, California, USA; b Department of Biochemistry, University of Cambridge, Cambridge, UK

**Keywords:** Ligand-gated ion channel, Cys-loop receptor, Serotonin receptor, Partial agonist, Binding site, Homology model

## Abstract

Antagonists, but not agonists, of the 5-HT_3_ receptor are useful therapeutic agents, and it is possible that partial agonists may also be potentially useful in the clinic. Here we show that 5-fluorotryptamine (5-FT) is a partial agonist at both 5-HT_3A_ and 5-HT_3AB_ receptors with an *R*_max_ (*I*_max_ / *I*_max_5-HT) of 0.64 and 0.45 respectively. It is about 10 fold less potent than 5-HT: EC_50_ = 16 and 27 μM, and *K*_i_ for displacement of [^3^H]granisetron binding = 0.8 and 1.8 μM for 5-HT_3A_ and 5-HT_3AB_ receptors respectively. We have also explored the potencies and efficacies of tryptamine and a range of 5-substituted tryptamine derivatives. At 5-HT_3A_ receptors tryptamine is a weak (*R*_max_ _= _0.15), low affinity (EC_50_ = 113 μM; *K*_i_ = 4.8 μM) partial agonist, while 5-chlorotryptamine has a similar affinity to 5-FT (EC_50_ _= _8.1 μM; *K*_i_ = 2.7 μM) but is a very weak partial agonist (*R*_max_ = 0. 0037). These, and data from 5-methyltryptamine and 5-methoxytryptamine, reveal the importance of size and electronegativity at this location for efficient channel opening.

## 1 Introduction

The 5-HT_3_ receptor is a member of Cys-loop family of ligand-gated ion channels, which also includes nicotinic acetylcholine, GABA and glycine receptors (Reeves and Lummis, 2002). These proteins are pentamers, and each subunit has a large extracellular N-terminal domain, four transmembrane helices (M1–M4) and an intracellular loop between M3 and M4. The binding site is located at the interface of two adjacent subunits and is formed by the convergence of three loops (A–C) from the principal subunit and another three loops (D–F) from the complementary subunit (Reeves and Lummis, 2002). Molecular details of the binding pocket have been extrapolated from the structure of the acetylcholine binding protein, which is homologous to the extracellular domain of Cys-loop receptors, and a range of amino acid residues that are important for agonist and antagonist binding have been identified (Reeves et al., 2003; Thompson et al., 2005). 5-HT_3_ receptors can function as homopentamers of 5-HT_3A_ receptor subunits, or as heteropentamers of 5-HT_3A_ and 5-HT_3B_ receptor subunits (5-HT_3AB_ receptors). The incorporation of B subunits results in some changes in the biophysical characteristics of the receptor, but has little effect on the pharmacological profile (Brady et al., 2001; Davies et al., 1999; Dubin et al., 1999).

5-HT_3_ receptor antagonists have been suggested to be potentially useful in treating inflammatory pain, anxiety, depression, schizophrenia, and drug abuse (Greenshaw and Silverstone, 1997), and are currently in clinical practice for the treatment of irritable bowel syndrome and emesis (Butler et al., 1988; Camilleri et al., 2000; Sanger and Nelson, 1989). It is therefore not surprising that many 5-HT_3_ receptor antagonists have been developed. There are, however, fewer 5-HT_3_ selective agonists. 2-methyl-5-HT and *m*CBPG have been widely used, and some novel compounds have been developed more recently such as benzoxazoles (Yoshida et al., 2005) and pyrroloquinoxaline-related compounds (Campiani et al., 1997). Here we explore the agonist properties of a compound closely related to 5-HT, 5-fluorotryptamine (5-FT), at both 5-HT_3A_ and 5-HT_3AB_ receptors, and compare them to the properties of 5-HT, *m*CBPG and tryptamine. We also explore several other 5-substituted tryptamine derivatives.

## 2 Materials and methods

### 2.1 Materials

**Fig. 1:**
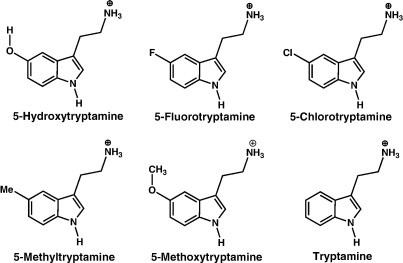
Structures of the 5-HT_3_ receptor agonists used in this study.

All cell culture reagents were obtained from Gibco BRL (Paisley, U.K.), except foetal calf serum which was from Labtech International (Ringmer, U.K.). [^3^H]granisetron (63.5 Ci mmol^− 1^) was from PerkinElmer (Boston, Massachusetts, USA). 5-FT, 5-chlorotryptamine (5-ClT), 5-methyltryptamine (5-MeT), 5-methoxytryptamine (5-MeOT) and tryptamine (Fig. 1) were from Sigma-Aldrich Co. Ltd. (Poole, Dorset, U.K.). All other reagents were of the highest obtainable grade.

### 2.2 Cell culture and oocyte maintenance

Human embryonic kidney (HEK) 293 cells were maintained in DMEM:F12 (Dulbecco's Modified Eagle Medium/Nutrient Mix F12 (1:1)) with GlutaMAX™ containing 10% foetal calf serum at 37 °C and 7% CO_2_ in a humidified atmosphere. *Xenopus laevis* oocyte positive females were purchased from NASCO (Fort Atkinson, Wisconsin, USA) and maintained according to standard methods (Goldin, 1992).

Harvested stage V–VI *Xenopus* oocytes were washed in six changes of ND96 (96 mM NaCl, 2 mM KCl, 1 mM MgCl_2_, 1.8 mM CaCl_2_, 5 mM HEPES, pH 7.5), de-folliculated in 1.5 mg ml^− 1^ collagenase Type 1A for approximately 2 h. Enzyme treatment was terminated by washing in six changes of ND96 and oocytes were stored in ND96 containing 2.5 mM sodium pyruvate, 50 mM gentamicin and 0.7 mM theophylline.

### 2.3 Receptor expression

Mouse 5-HT_3A_ (accession number: AY605711) or 5-HT_3B_ (accession number: NM_020274, kindly provided by Ewen Kirkness) subunit cDNAs were cloned into pGEMHE for oocyte expression (Liman et al., 1992). cRNA was in vitro transcribed from linearised (NheI) plasmid cDNA template using the mMessage mMachine T7 kit (Ambion, Austin, Texas, USA). Stage V and VI oocytes were injected with 50 ng–100 ng cRNA, and recorded from 1–4 days post-injection. For expression in HEK 293 cells, 5-HT_3_ receptor subunit cDNAs were cloned into pcDNA3.1 (Invitrogen Ltd., Paisley, UK.). Mutagenesis reactions were performed using the Kunkel method and confirmed by DNA sequencing. Cells were transfected using calcium phosphate precipitation at 80–90% confluency (Jordan et al., 1996). Following transfection cells were incubated for 3–4 days before assay.

### 2.4 Radioligand binding

This was undertaken in HEK 293 cells which provide an established and robust method of studying ligand binding. Methods were as previously described (Lummis et al., 1993), with minor modifications. Briefly, transfected HEK 293 cells were washed twice with phosphate buffered saline (PBS) at room temperature and scraped into 1 ml of ice-cold HEPES buffer (10 mM, pH 7.4) containing the following proteinase inhibitors (PI): 1 mM EDTA, 50 μg ml^− 1^ soybean trypsin inhibitor, 50 μg/ml bacitracin and 0.1 mM phenylmethylsulphonyl fluoride. After thawing, they were washed with HEPES buffer, resuspended, and 50 μg of cell membranes incubated in 0.5 ml HEPES buffer containing 0.5 nM [^3^H]granisetron (a concentration approximately equivalent to the *K*_d_); non-specific binding was determined using 10 μM quipazine. Competition binding was performed using ligand concentrations from 0.1 μM–10 mM. Reactions were incubated for at least 1 h at 4 °C and terminated by vacuum filtration using a Brandel cell harvester onto GF/B filters pre-soaked in 0.3% polyethyleneimine. Radioactivity was determined by scintillation counting using a Beckman LS6000SC (Fullerton, California, USA). Competition binding data were analyzed by iterative curve fitting (GraphPad Prism v3.02, GraphPad Software, San Diego, California, USA), according to the equation:

$$y=B_{\text{min}}+\frac{B_{\text{max}}-B_{\text{min}}}{1+10^{\left[L\right]-\log{\mathrm{IC}}_{\text{50}}}}$$

where *B*_min_ is the lowest total binding, *B*_max_ is the maximum specific binding at equilibrium, [*L*] is the concentration of competing ligand and IC_50_ is the concentration of competing ligand that blocks half of the specific bound radioligand. *K*_i_ values were estimated from IC_50_ values using the Cheng–Prusoff equation:

$$K_{\text{i}}=\frac{{\mathrm{IC}}_{50}}{1+\left[L\right]/K_{\text{d}}}$$

where *K*_i_ is the equilibrium dissociation constant for binding of the unlabeled antagonist, [*L*] is the concentration of radioligand and *K*_d_ is the equilibrium dissociation constant of the radioligand.

### 2.5 Electrophysiology

Agonist-induced currents were recorded at 22–25 °C from individual oocytes using the OpusXpress system (Molecular Devices Axon Instruments, Union City, CA). 5-HT, *m*-chlorophenylbiguanide (*m*CPBG), 5-FT and tryptamine (Sigma) were stored as 20–100 mM aliquots at−20 °C, diluted in Ca-free ND96 buffer (96 mM NaCl, 2 mM KCl, 1 mM MgCl_2,_ 5 mM HEPES, pH 7.5) and delivered to cells via the automated perfusion system of the OpusXpress. Glass microelectrodes were backfilled with 3 M KCl and had a resistance of ∼ 1 MΩ. The holding potential was − 60 mV. To determine EC_50_ values, concentration–response data were fitted to the four-parameter logistic equation, *I* = *I*_min_ + (*I*_max_−*I*_min_) / (1 + 10 ^(log(EC50 − [*A*])*n*_H_^), where *I*_max_ is the maximal response plateau, *I*_min_ is the minimum response plateau, [*A*] is the log concentration of agonist and *n*_H_ is the Hill coefficient, using PRISM v4. 03 software (GraphPad, San Diego, CA). Relative efficacies of the partial agonists are reported as *R*_max_ = *I*_max_drug / *I*_max_5-HT. One-way ANOVAs were performed with Dunnett's post test to determine statistical significance. Data are quoted as mean ± SEM (*n*) unless otherwise stated.

## 3 Results

### 3.1 Effects of agonists on 5-HT_3_ receptor mediated currents

**Fig. 2:**
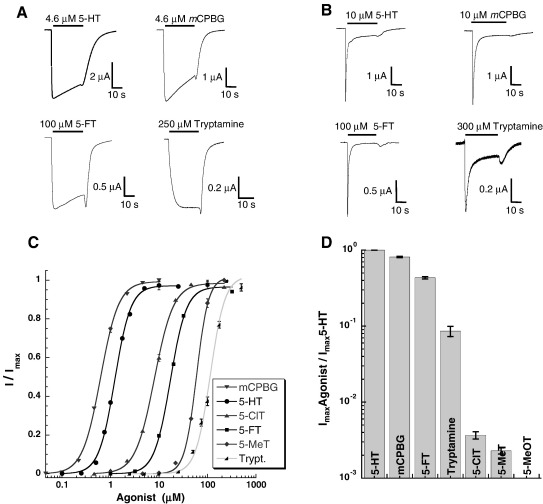
Properties of 5-HT_3A_ and 5-HT_3AB_ receptors expressed in *Xenopus* oocytes. Typical responses to maximal concentrations of 5-HT, mCPBG, 5-FT and tryptamine in (A) 5-HT_3A_ and (B) 5-HT_3AB_ receptors; (C) Concentration–response curves in 5-HT_3A_ receptors; (D) Relative efficacies (*R*_max_) of agonists compared to 5-HT.

Application of 5-HT to *Xenopus* oocytes expressing 5-HT_3A_ or 5-HT_3AB_ receptors produced concentration-dependent, rapidly activating, inward currents that desensitised over the time-course of the application (Fig. 2). Plotting current amplitude against a series of 5-HT concentrations revealed EC_50_s of 1.4 μM and 3.2 μM with Hill slopes of 2.5 and 1.4 respectively (Table 1).

Application of 5-FT to *Xenopus* oocytes expressing 5-HT_3A_ or 5-HT_3AB_ receptors also produced concentration-dependent, rapidly activating, inward currents, with EC_50_s of 16 μM and 27 μM and Hill slopes of 2.4 and 1.4 respectively. A maximal concentration of 5-FT, however, did not elicit the same maximal currents as those obtained from 5-HT application in the same oocyte, indicating a partial agonist; 5-FT had a *R*_max_ (*I*_max_ drug / *I*_max_ 5-HT) of 0.64 ± 0.03 for 5-HT_3A_ receptors and *R*_max_ of 0.45 ± 0.04 for 5-HT_3AB_ receptors (Table 2).

Application of *m*CPBG produced concentration-dependent, rapidly activating, inward currents, with EC_50_s of 0.5 μM and 1.1 μM and Hill slopes of 2.3 and 1.6 for 5-HT_3A_ or 5-HT_3AB_ receptors, respectively. This compound had an *R*_max_ of 0.74 ± 0.07 for 5-HT_3A_ receptors and 0.92 ± 0. 09 for 5-HT_3AB_ receptors.

Application of tryptamine produced concentration-dependent, rapidly activating, inward currents, but here there was little desensitisation over the time-course of the application (Fig. 2). Plotting current amplitude against a series of tryptamine concentrations revealed EC_50_s of 113 μM and 61 μM with Hill slopes of 2.5 and 1.8 for 5-HT_3A_ and 5-HT_3AB_ receptors respectively. Tryptamine had an *R*_max_ of 0.15 ± 0. 06 for 5-HT_3A_ receptors and an *R*_max_ of 0.14 ± 0. 03 for 5-HT_3AB_ receptors.

5-ClT was a very weak partial agonist of 5-HT_3A_ receptors, with an *R*_max_ of 0. 0037; the size of the responses precluded data from 5-HT_3AB_ receptors. Despite its low *R*_max_, 5- ClT had an EC_50_ (8.1 ± 0.3 μM, *n* = 11) that was lower than that of 5-FT (16 μM).

5-MeT was also a very weak partial agonist at 5-HT_3A_ receptors with an *R*_max_ of 0. 0023. Dose response curves yielded an EC_50_ of 60 ± 3μM (*n* = 3) indicating it was slightly more potent than tryptamine (EC_50_ = 113 μM).

5-MeOT was unable to activate 5-HT_3_ receptors at concentrations up to 10 mM.

### 3.2 [^3^H]granisetron binding studies

Saturation binding studies revealed no significant difference in the affinity (*K*_d_) of [^3^H]granisetron between 5-HT_3A_ and 5-HT_3AB_ receptors (0.42 ± 0.15 and 0.62 ± 0.21 nM respectively, *n* = 3). Competition binding studies using [^3^H]granisetron revealed displacement of specific binding in a concentration-dependent manner by all the ligands. *K*_i_s (Table 3) revealed that 5-HT, *m*CPBG, 5FT and tryptamine did not substantially distinguish between 5-HT_3A_ and 5-HT_3AB_ receptors.

[^3^H]granisetron competition studies using 5-ClT, 5-MeT and 5-MeOT on membranes from cells expressing 5-HT_3A_ receptors revealed 5-ClT had a similar *K*_i_ to 5-FT, which was ∼ 10 fold more than the *K*_i_ for 5-HT. Values for tryptamine, 5-MeT and 5-MeOT were ∼ 50, 100 and 300 fold greater than 5-HT respectively (Table 3).

Competition radioligand binding studies on the mutant receptors N128A, T181A and E236A, revealed no significant changes in *K*_i_ values compared to WT receptors for either 5-FT or 5-HT (Table 4). E129A and T179A mutant receptors had either no specific radioligand binding, or levels were too low to obtain accurate data as previously reported (Sullivan et al., 2006).

## 4 Discussion

The data described here show that 5-FT is a partial agonist at both 5-HT_3A_ and 5-HT_3AB_ receptors, with an *R*_max_ close to 0.5 and an EC_50_ about 10 fold higher than 5-HT. Similarly, tryptamine is a partial agonist at both types of receptor, as previously reported for various native and recombinant 5-HT_3_ receptors, including those natively expressed in N1E-115 cells, which may possess both 5-HT_3A_ and 5-HT_3B_ receptor subunits (van Hooft and Vijverberg, 1996). Tryptamine has a lower potency than both 5-HT and 5-FT (EC_50_ 10–100 fold higher) and a lower *R*_max_, indicating the importance of the group at the 5 position of 5-HT. Further studies on other 5-substituted tryptamine derivatives confirm this hypothesis, and also reveal the importance of size and electronegativity at this location for efficient channel opening.

Subtle differences between 5-HT_3A_ and 5-HT_3AB_ receptors have been reported by a number of authors, and were also observed in the current study. Compared to the 5-HT_3A_ receptor, responses from 5-HT_3AB_ receptors are smaller and desensitise more rapidly; EC_5O_ and *K*_d_ values differ by ∼ 2 fold and there is an ∼ 2 fold decrease in the Hill slope of the dose response curves. There is also a difference in the efficacy of *m*CPBG, which acts as a partial agonist at 5-HT_3A_ receptors, but a full agonist at 5-HT_3AB_ receptors. This indicates gating characteristics of the two receptors are different, and indeed it has been established that the channel conductance is greatly increased in 5-HT_3AB_ receptors (Davies et al., 1999).

Previous functional studies have revealed only small differences in the affinities (EC_50_ and IC_50_s) of 5-HT_3A_ and 5-HT_3AB_ receptors for a range of 5-HT_3_ selective ligands (Brady et al., 2001), and we observed a similar absence of selectivity for 5-HT, *m*CPBG, 5-FT and tryptamine in this study. These results are somewhat surprising, given that a recent study has suggested that in the heterologously expressed 5-HT_3AB_ receptors the subunits are in the order BABBA (Barrera et al., 2005), and, as agonist binding sites in Cys-loop receptors are constituted from two adjacent subunits, these data imply that binding interfaces would either be AB (most likely), BA or BB.

**Fig. 3:**
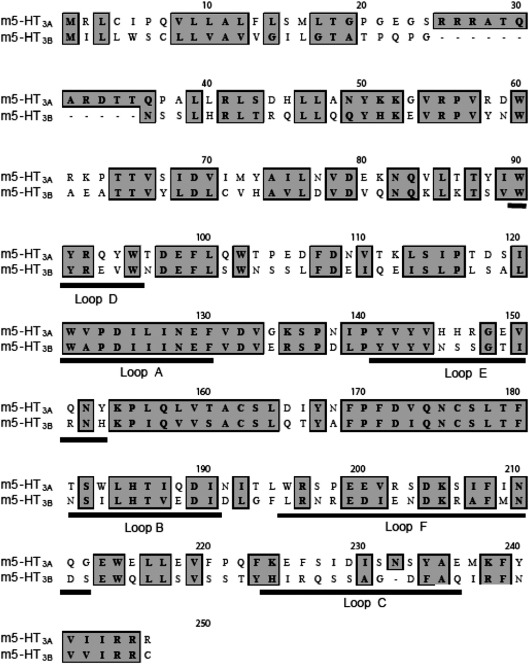
Alignment of 5-HT_3A_ and 5-HT_3B_ subunit sequences. Residues that have similar chemical properties are shown in grey. The binding loops that constitute the binding site are underlined.

Based on the sequence alignment (Fig. 3), one would expect significant structural differences due to the different residues that would contribute to AA compared to AB/BA or BB binding sites. At present, we cannot explain why there are not larger changes in pharmacological characteristics of the AB receptor.

**Fig. 4:**
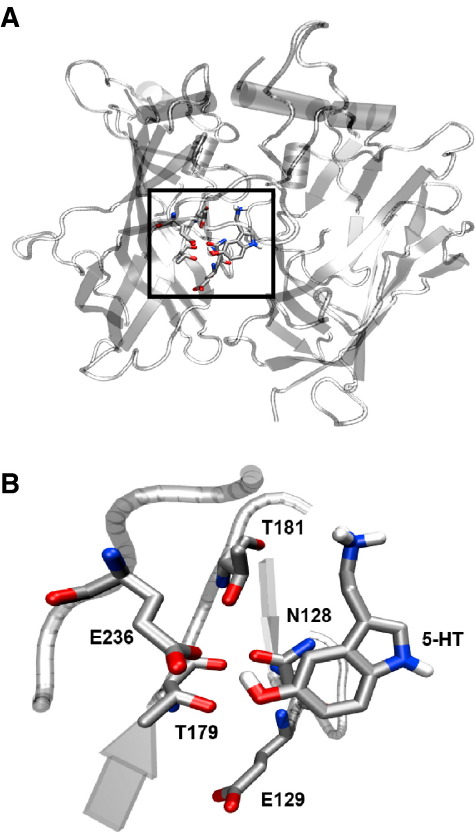
5-HT docked into a homology of the 5-HT_3_ receptor (Reeves et al., 2003). A. The extracellular domains of two subunits of the 5-HT_3_ receptor showing the location of the binding pocket (boxed) at their interface. B. Enlarged image of the binding site showing the proximity of the hydroxyl group of 5-HT to the hydrophilic residues Asn128, Glu129 Thr179, Thr181 and Glu236.

The new data reveal some interesting features of the binding pocket. Tryptamine is ∼ 100 fold less potent and much less efficacious than 5-HT (*R*_max_ _=_ ∼ 0.15), establishing the importance of the hydroxyl group. However 5-FT can significantly compensate for the lack of a hydroxyl; it is only 10 fold less potent than 5-HT and *R*_max_ = ∼ 0.5. In our model of the binding pocket (Reeves et al., 2003), the hydroxyl of 5-HT is located in a hydrophilic pocket constituted of Asn128, Glu129, Thr179, Thr181 and Glu236, and it has the potential to hydrogen bond with at least one of these residues (Fig. 4). Mutation of Asn128, Thr181 and Glu236 to Ala results in no significant changes to the 5-HT *K*_i_, suggesting that Glu129 and Thr179 are the most likely residues to contribute to hydrogen bonds. However as alanine substitutions at these positions result in poor receptor expression we cannot yet prove this hypothesis. 5-FT can be located in a similar location to 5-HT, but we believe it is unlikely that F also forms hydrogen bonds here. Fluorine is the most electronegative element, and as such it is reluctant to donate a lone pair of electrons to a hydrogen bond donor. As a result, organic fluorine (fluorine bonded to a carbon) hardly ever accepts a hydrogen bond (Dunitz, 2004). Even without a hydrogen bond, however, it appears that an electronegative atom is more favourable than no substituent at all at this location.

To further explore this region of the binding site, we examined 5-ClT, 5-MeT and 5-MeOT in 5-HT_3A_ receptors. 5-ClT was of similar potency to 5-FT in the functional assays (EC_50_ = 8 μM) but was much less effective in opening the channel (*R*_max_ = 0. 0037). 5-ClT and 5-FT bind to the receptor with similar affinities (*K*_i_s are not significantly different), demonstrating there is no relationship between *K*_i_ or EC_50_ and *R*_max_ Thus it appears that the atom at the 5 position of tryptamine plays a critical role in the conformational changes that result in channel opening. Since both 5-FT and 5-ClT present a relatively electronegative atom at this position, we propose that the increased steric size of Cl vs. F contributes to decreased efficacy of 5-ClT. Sterics also rationalize the inefficacy of 5-MeOT, which has an electronegative element in the 5 position but is apparently too large. The data from 5-MeT also support the hypothesis that size and polarity are important; Me is a similar size to Cl, but is nonpolar, and 5-MeT is less effective at opening the channel.

The data also show that for most agonists there is a direct relationship between EC_50_ and *K*_i,_ with EC_50_s 13–50 fold higher than *K*_i_. This is expected, as *K*_i_ values are considered to represent binding to a high affinity desensitised state. However, for 5-ClT and 5-MeT, which have very low efficacy, EC_50_ is less than 5 fold higher than *K*_i_. This suggests that if agonist binding does not result in significant channel opening (*R*_max_ less than 0.01), then there may be no significant entry of receptors into a high affinity state.

Partial agonists are increasingly being used to distinguish between binding and gating events at Cys-loop receptors, and 5-FT, with an *R*_max_ of ∼ 0.5, will be a useful addition to this class of compounds which includes the more usually used *m*CBPG (*R*_max_ = ∼ 0.8) and 2-methyl-5-HT (*R*_max_ = ∼ 0.2). Partial agonists are also potentially useful as therapeutic agents. The most well-established role of 5-HT_3_ receptors is in regulating gastrointestinal motility and the vomiting reflex, although they may play a role in many other neuronal functions. Currently, 5-HT_3_ receptor antagonists are used clinically as anti-emetics, and to treat irritable bowel syndrome (Butler et al., 1988; Camilleri et al., 2000; Sanger and Nelson, 1989). However, there is some evidence that these compounds also cause side effects in many patients, by inhibiting normal lower bowel function (Talley, 1992). Thus there has been an increased interest in 5- HT_3_ receptor partial agonists which might control gastroenteric motility without completely blocking 5-HT_3_-sensitized nerve function (Sato et al., 1997; Sato et al., 1998). 5-HT_3_ receptor agonists also have a potential therapeutic role as they can modulate acetylcholine release in vivo (Consolo et al., 1994), making these compounds of interest for the treatment of neurodegenerative and neuropsychiatric disorders in which cholinergic neurons are affected. Full 5-HT_3_ receptor agonists, however, cause nausea and vomiting; thus partial agonists are potentially more useful for therapeutic applications in this area. Recently developed compounds, e.g those described by Yoshida et al. (2005), are probably potentially more useful as therapeutics than 5-FT, but a comparison of their actions compared to 5-FT may clarify details of their mode(s) of action.

In conclusion we have shown that 5-FT is a partial agonist at both homomeric 5-HT_3A_ and heteromultimeric 5-HT_3AB_ receptors. The data have also revealed that the atom in the 5 position of 5-HT plays an important role both in receptor binding and in subsequent channel gating.

## Figures and Tables

**Table 1:** Functional parameters of 5-HT_3A_ and 5-HT_3AB_ receptors

	*p*EC_50_	EC*_5_*_0_ (μM)	*n*_H_
A 5-HT	5.85 ± 0.10	1.4	2.5 ± 0.4
AB 5-HT	5.49 ± 0. 03	3.2	1.4 ± 0.4
A 5-FT	4.80 ± 0. 05	16	2.4 ± 0.5
AB 5-FT	4.57 ± 0. 08	27	1.4 ± 0.3
A *m*CPBG	6.29 ± 0. 04	0.5	2.3 ± 0.4
AB *m*CPBG	5.96 ± 0. 06	1.1	1.6 ± 0.4
A tryptamine	3.91 ±.03	113	2.5 ± 0.5
AB tryptamine	4.22 ±.09	61	1.8 ± 0.5

Data = mean ± SEM, *n* = 4–6.

**Table 2:** Relative efficacies (*R*_max_ = *I*_max_ drug / *I*_max_ 5-HT)

	5-FT	*m*CPBG	Tryptamine
A	0.64 ± 0.03	0.74 ± 0.07	0.15 ± 0.06
AB	0.45 ± 0.04	0.92 ± 0.09	0.14 ± 0.02

Data = mean ± SEM, *n* = 5–9.

**Table 3:** Inhibition constants derived from [^3^H] granisetron binding to 5-HT_3A_ and 5-HT_3AB_ receptors

	A (*K*_i_, μM)	AB (*K*_i_, μM)
5-HT	0.11 ± 0.02	0.11 ± 0.03
*m*CPBG	0.010 ± 0.003	0.012 ± 0.004
5-FT	0.83 ± 0.17	1.8 ± 0.4
Tryptamine	4.8 ± 0.9	15.5 ± 3.5
5-Cl-tryptamine	2.7 ± 0.7	3.1 ± 1.1
5-Me-tryptamine	11. 0 ± 0.9	7.7 ± 1.1
5-MeO-tryptamine	34.9 ± 3.0	21.7 ± 2.1

Data = mean ± SEM, *n* = 3–6.

**Table 4:** Inhibition constants derived from [^3^H] granisetron binding to mutant 5-HT_3A_ receptors

	5-HT (*K*_i_, μM)	5-FT (*K*_i_, μM)
WT	0.11 ± 0.02	0.83 ± 0.17
N128A	0.21 ± 0.05	2.43 ± 0.47
T181A	0.19 ± 0.04	0.02 ± 0.34
E236A	0.20 ± 0.05	1.62 ± 0.41

Data = mean + SEM, *n* = 3–6.
